# A Cross-Sectional Investigation of Chronic Exposure to Microcystin in Relationship to Childhood Liver Damage in the Three Gorges Reservoir Region, China

**DOI:** 10.1289/ehp.1002412

**Published:** 2011-05-11

**Authors:** Yan Li, Ji-an Chen, Qing Zhao, Chaowen Pu, Zhiqun Qiu, Renping Zhang, Weiqun Shu

**Affiliations:** 1 Department of Environmental Hygiene, College of Preventive Medicine, Third Military Medical University, Chongqing, China; 2 The Center for Disease Control and Prevention in Fuling Borough, Chongqing, China

**Keywords:** childhood, chronic exposure, cross-sectional investigation, liver damage, microcystin, serum enzyme, total daily intake

## Abstract

Background: Microcystin-producing *Microcystis* bloom is a severe water problem in the world. Some reports indicate that chronic exposure to microcystin may result in liver damage in adults, but information on effects in children is limited.

Objective: We investigated the relationship between microcystin exposure and liver damage in children.

Methods: We measured microcystin concentrations in drinking water and aquatic food (carp and duck) from two lakes and four wells. Participants were 1,322 children 7–15 years of age who obtained drinking water from one of the tested sources, completed questionnaires, and provided blood samples for serum liver enzymes [alanine aminotransferase (ALT), aspartate aminotransferase (AST), alkaline phosphatase (ALP) and γ-glutamyltransferase (GGT)] and serum microcystin analysis. Multivariable logistic regression was used to identify risk factors associated with liver damage (two or more abnormal serum enzyme levels in ALT, AST, ALP, or GGT).

Results: Microcystin was detected in most samples of water and aquatic food from two lakes. Children who drank water from the lake with the highest microcystin concentrations had a total estimated daily microcystin intake of 2.03 μg, a value much higher than the tolerable daily intake (0.40 μg) proposed by the World Health Organization for children. Hepatitis B virus (HBV) infection, use of hepatotoxic medicines, and microcystin exposure were associated with liver damage. AST and ALP levels were significantly higher in high-microcystin-exposed children than in low-exposed children and unexposed children when participants who were HBV-positive or hepatotoxic medicine users were excluded from the analysis.

Conclusion: These results suggest that chronic exposure to microcystin may be associated with liver damage in children in the Three Gorges Reservoir Region.

In recent years, the growth of aquaculture production systems, mostly the fishing kind, coupled with a lack of control has led to an increase of eutrophication and cyanobacteria blooms (Chen et al. 2009b). Cyanobacteria blooms have been reported as a severe water problem in many freshwater lakes of the world, especially in the Chongqing area of the Three Gorges Reservoir Region in China (Pu et al. 2007). Blooms can affect the water quality, as indicated by changes in pH, transparency, and biodiversity, and produce a variety of toxins including microcystin, nodularin, and anatoxina. Microcystin, the most common and thoroughly studied toxin (Gilroy et al. 2000), belongs to a family of cyclic hepatotoxic heptapeptides produced by cyanobacteria from the genera *Microcystis*, *Oscillatoria*, *Anabaena*, and *Nostoc* (Rivasseau et al. 1998). To date, > 80 microcystin isoforms have been isolated and identified (Gupta et al. 2003), and microcystin-LR (MC-LR) is the most common and toxic variant (Dietrich and Hoeger 2005). The liver is the primary target of microcystin. Animal experiments have shown that chronic exposure to microcystin affects liver histology and function (Beasley et al. 2000) and may cause liver cancer with long-term exposure (Grosse et al. 2006).

Animal exposure via drinking water contaminated with cyanobacteria was documented at least 130 years ago (Francis 1878). Since then, people have become increasingly concerned about this issue (Backer et al. 2009; Pilotto et al. 1999). Zhang et al. (2009) reported that microcystin concentrations were the highest in the liver and gut of phytoplanktivorous fish, followed by omnivorous fish, and were the lowest in carnivorous fish. More important, the microcystin concentrations in the fish mentioned above exceeded the tolerable daily intake (TDI) proposed by the World Health Organization (WHO) in adults and in children (WHO 1999). Extensive laboratory studies have concluded that chronic exposure to microcystin can cause liver cell damage and ultimately lead to a significant increase in serum liver enzyme levels (Carmichael et al. 2001; Zhang et al. 2008). Falconer et al. (1983) linked toxic algae in a water supply reservoir to serum enzyme elevations in the people exposed to microcystin. In Caruaru, Brazil, 76 patients died because of dialysate contamination by microcystin (Carmichael et al. 2001). Ueno et al. (1996) hypothesized that the high incidence of primary liver cancer in southeast China is likely related to microcystin contaminations in drinking water. Chen et al. (2009a) identified microcystin in the serum of highly exposed Brazilian fishermen as well as indication of liver damage.

Although acute and short-term hepatotoxic effects have been partially documented in humans after exposure to high microcystin levels, effects of chronic exposure to low microcystin levels are unclear, especially in children. The objectives of this study were to *a*) compare serum enzyme levels between two exposed groups and one unexposed group of children; *b*) determine whether microcystin exposure is a risk factor for liver damage, using multivariable logistic regression analysis; and *c*) measure microcystin levels in the serum samples and assess the relationship between chronic microcystin exposure and liver damage in children.

## Materials and Methods

This study was approved by the Institutional Review Board of Third Military Medical University and the Center for Disease Prevention and Control (CDC) in Fuling Borough. We have complied with all applicable requirements for the protection of participants. The parents of participants gave written informed consent before the study.

*Site selection.* Three drinking water sources for local residents of the Three Gorges Reservoir Region were selected for sampling: *a*) community wells, which are rarely contaminated by microcystin; *b*) a lake that has occasional cyanobacterial blooms (lake 1), and *c*) a lake that had regular occurrences of cyanobacterial blooms over the previous 5 years (lake 2) (Pu and Han 2004; Pu et al. 2003). Microcystin surveillance data are used to select low-, medium-, and high-exposure water sources.

*Study participants.* Potential study participants (*n* =1,441) were randomly selected from five schools. Participants were classified as having low exposure and high exposure if they consumed water and aquatic food for > 5 years from lake 1 or lake 2, respectively, and as unexposed if they drank well water for > 5 years and rarely consumed fish or duck from lake 1 or lake 2.

*Water sample collection and microcystin analysis.* Lake water samples were collected from the major axis of the two lakes, and well water samples were collected from community wells in the study location. Water was sampled once a day (in the morning) over 6 days (8–13 August) each year from 2005 to 2009. Presterilized Plexiglas water samplers (Chinese Academy of Sciences, Beijing) were used to collect 5-L water samples from 0.5 m below the surface.

ELISA kits [MC-LR monoclonal antibody (MC8C10), Chinese Academy of Sciences, Beijing, China] and the Anthos 2010 Automatic ELISA analyzer (Anthos, Wals, Austria) were used to measure the intracellular and extracellular microcystin levels. The intracellular microcystin was extracted from cyanobacterial cells using the method described by Tsuji et al. (1994). The water samples and microcystin standard (0, 0.25, 0.5, 1, 2, 4 μg/L) were added to a 96-well plate coated with MC-LR bovine serum albumin conjugate. The MC-LR monoclonal antibody solution was added into the wells and incubated for 50 min at room temperature. After washing, the horseradish peroxidase-labeled goat anti-mouse IgG (TAGO 4550) plus substrate solution was added and incubated for 40 min. Finally, color solutions were added and incubated for 10 min, and absorbances were read at 450 nm using the ELISA photometer. The limit of detection (LOD) of this method was 0.1 μg/L, and the cross-reaction rate (percent) to other microcystin types (microcystin-YR and microcystin-RR) was < 10%. To verify the accuracy of the ELISA method, the water samples that were collected in 2009 were sent to Chongqing Environmental Monitoring Center for reanalysis by high-performance liquid chromatography using the methods of Lawton et al. (1994).

*Aquatic food collection and microcystin analysis.* In August 2009, five types of carp—*Hypophthalmichthys molitrix, Aristichys nobilis, Carassius auratus, Cyprinus carpio*, and *Ctenopharyngodon idellus—*and one species of duck were collected from lake 1 and lake 2 for microcystin analysis. After sacrifice, the livers and muscles of the fish and duck were dissected, measured, weighed, and frozen immediately at –80°C.

To determine microcystin levels, all liver and muscle samples were lyophilized using an Alpha 2-4 Freeze Dryer (Martin Christ, Osterode, Germany). The lyophilized samples were extracted and purified following the methods of Magalhaes et al. (2001) with a slight modification. Each sample was treated with 100% methanol and hexane. The obtained methanolic fraction was eluted in a C-18 cartridge, washed, then eluted in 30 mL 20% methanol and 50 mL 100% methanol. The methanol fraction was dried and re-dissolved in 1.0 mL 50% methanol. The suspension was filtered through a nylon filter (0.45 μm) and stored at −20°C. The microcystin detection method for microcystin in livers and muscles was same as that in the water samples.

*Study questionnaire.* We also investigated the risk factors related to liver damage, such as microcystin exposure and strenuous exercise. With the assistance of the students’ parents, we administered the questionnaire to each student. Students were shown a 0.5-L and a 1-L beaker and asked “How many glasses of water or soup as measured by such a glass do you drink every day?” and “How many times do you consume fish or duck a week and how much of this food do you consume each time?” Students were classified as using hepatotoxic medications if they reported daily use for at least 3 months of medications defined as hepatotoxic according to the Council for International Organizations of Medical Sciences (Miren et al. 2011), including antituberculosis drugs (e.g., isoniazid, rifampicin, sodium aminosalicylate), anticancer drugs (e.g., amethopterin, mercaptopurine, asparaginase), antithyroid drugs (e.g., carbimazole, thiamazole, thiouracil), antibiotics (e.g., chloromycetin, erythromycin, mydecamycin), and antipyretic analgesics (Farrell 1997). According to the American College of Sports Medicine, strenuous exercise is defined as exercise that exceeds 6 times the respiratory quotient of the body’s load, for example, running > 30 min or 2 km, playing basketball for 40 min, or doing aerobics for 40 min (Malinoski 1992). We asked students “Did you undertake running more than 30 min or playing basketball for 40 min the day before the survey?”

*Blood sample collection and serum enzyme analysis.* A venipuncture system was used by trained nurses at The Southwest Hospital of Chongqing to collect blood samples from students in the morning prior to each student’s first meal. Approximately 8 mL blood per participant was drawn into tubes containing EDTA. Serum was isolated, and alanine aminotransferase (ALT), aspartate aminotransferase (AST), alkaline phosphatase (ALP), and γ-glutamyltransferase (GGT) were assayed using Synchron Clinical System LX20 (Beckman-Coulter Diagnosis, Fullerton, CA, USA). Normal ranges of ALT, AST, GGT, and ALP are defined as 7–40 U/L, 8–40 U/L, 4–50 U/L, and 130–560 U/L, respectively. Liver damage was defined as two consecutively abnormal serum enzyme detections. Serum hepatitis Bs antigen, hepatitis Bs antibody, hepatitis Be antigen, hepatitis Be antibody, and hepatitis Bc antibody were detected using an ELISA kit (Hepanostika Uni-form, Organon Teknika B.V., Boxtel, the Netherlands) and performed according to the instructions.

*Serum microcystin level analysis.* Each serum sample (300 μL) was lyophilized and extracted with 1,100 μL analytical-grade acetone twice. The combined extract was centrifuged at 20,000 × *g* for 20 min; the supernatant was mixed with an equal volume of hexane three times. After precipitation, the hexane layer was removed and the precipitation was blown dry by nitrogen gas at 60°C, and then dissolved in deionized water. The dissolved solution was passed through an Oasis hydrophilic-lipophilic balance solid phase extraction cartridge (2 g/12 mL; Waters, Milford, MA, USA) that had been preconditioned with 10 mL 100% methanol (MeOH) and 10 mL deionized water. The cartridge was washed with 10 mL 100% MeOH and 10 mL deionized water; the elution was blown dried again, then the sample solution was dissolved in 100% MeOH (1.0 mL) and analyzed for microcystin using ELISA.

*Microcystin recovery test.* To confirm the reliability of the ELISA results, a recovery test with added microcystin standard material in samples was conducted. Each sample was measured three times. The average recovery from water samples was 92.0%, and the relative standard deviation (RSD) was between 5.4% and 8.1%. For aquatic food samples, average recovery was 89.0%, with RSD between 3.4% and 11.0%. The average recovery from serum samples was 84.7%, with RSD between 2.8% and 12.6%.

*Statistical analyses.* Data were analyzed with Microsoft Excel 2003 (Microsoft Corporation, Redmond, WA, USA) and SPSS (SPSS 13.0; StatSoft Inc., Tulsa, OK, USA). Subject characteristics and serum enzymes were compared using the Kruskal–Wallis test and chi-square tests. A logistic regression analysis was used to explore the relationship between microcystin exposure (unexposed, exposed) and liver damage (dichotomous based on two or more abnormally elevated liver enzyme assays). Other model covariates included body mass index (BMI) (categorical, < 16, 16–18, > 19); hepatitis B virus (HBV) infection (yes or no); hepatotoxic medicine use (yes or no); strenuous exercise the previous day (yes or no); passive smoking (yes if exposed to environmental smoke > 15 min/day and > 1 day/week; no otherwise).

## Results

*MC-LR concentrations in drinking water and aquatic food.* MC-LR was detected in almost all water samples collected from lake 1 and lake 2 (Table 1). The highest mean (± SD) concentration was 4.3 ± 1.0 μg MC-LR equivalents per liter (MC-LReq/L) in samples collected from lake 2 in 2008. The mean microcystin concentrations for all samples collected during the study were 0.24 μg MC-LReq/L for lake 1 and 2.58 μg MC-LReq/L for lake 2. MC-LR was detected in only one well water sample in 2008; all other well water samples were under the limits of detection using ELISA.

**Table 1:** MC-LR levels in three water sources in the Three Gorges Reservoir Region.

Well water*b*					Lake 1 water*b*					Lake 2 water*b*				
Year	*n*	Mean ± SD	Range	*n*	Mean ± SD	Range	*n*	Mean ± SD	Range
2005*c*		4		—		< LOD		4		0.2 ± 0.2		0.1–0.5		4		1.1 ± 0.3		0.8–1.5
2006*c*		6		—		< LOD		3		0.1 ± 0.2		< LOD–0.3		3		3.2 ± 1.7		1.8–5.1
2007*c*		4		—		< LOD		4		0.4 ± 0.3		0.2–0.8		4		4.1 ± 1.6		2.3–6.1
2008*c*		8		0.1 ± 0.1		< LOD–0.2		4		0.3 ± 0.2		0.1–0.6		4		4.3 ± 1.0		3.5–5.7
2009*c*		5		—		< LOD		5		0.2 ± 0.2		0.1–0.5		5		0.2 ± 0.1		0.1–0.4
2009*d*		5		—		< LOD		5		0.3 ± 0.2		0.1–0.6		5		0.4 ± 0.2		0.3–0.7
Average*c*		—								0.24 ± 0.11						2.58 ± 1.86		
**a**Water samples were collected daily from 8 to 13 August 2005–2009. LOD = 0.1 μg/L. **b**Micrograms MC-LReq /L. **c**Detected by ELISA. **d**Detected by high-performance liquid chromatography.																		

**Figure 1:**
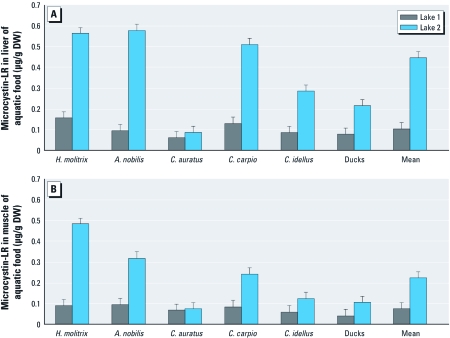
(*A*) MC-LR concentrations in the liver of aquatic food from lake 1 and lake 2. (*B*) MC-LR concentrations in the muscle of aquatic food from lake 1 and lake 2. DW, dry weight. All samples were collected in August 2009.

MC-LR was detected in all of the aquatic food samples, with significantly higher concentrations in liver samples than muscle samples, and higher concentrations in both liver and muscle samples from fish and ducks collected from lake 2 compared with samples from lake 1 (Figures 1A and 1B). The concentrations of MC-LR in liver samples varied between different fish, with the highest levels in *A. nobilis* [0.580 ± 0.034 μg/g dry weight (DW)], followed by *H. molitrix* (0.564 ± 0.025 μg/g DW) and *C. carpio* (0.510 ± 0.028 μg/g DW). The mean microcystin concentration in muscle (the major edible parts) of the fish and ducks from lake 1 and lake 2 were 0.10 µg/g and 0.22 µg/g, respectively.

**Figure 2:**
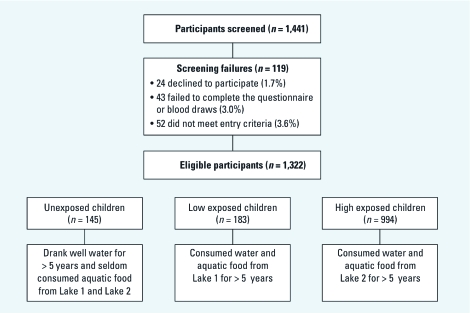
The screening process. Participants were randomly selected from five schools in Fuling Borough, Chongqing.

*Subject characteristics.* To be eligible as a study participant, the student had to be between 7 and 15 years of age; have consumed water and aquatic food (local fish and duck) in the study area for > 5 years, with no more than 3 months spent outside of the area during that time; and had to be able to complete the entire questionnaire and blood sample collection. Exclusion criteria included present or previous diagnosis of severe chronic disease, except liver disease, and long-term toxic exposure history based on information provided by the participants’ parents. Among 1,441 recruited children, 24 (1.7%) declined to participate, 43 (3.0%) failed to complete the questionnaires or blood draws, and 52 (3.6%) did not meet the study inclusion criteria. Ultimately, a total of 1,322 participants completed all steps of the study (Figure 2). Most of the children were classified as high exposed (75%, compared with 14% low exposed and 11% unexposed) (Table 2). High-exposed children were older than low-exposed or unexposed children (average age 11.6, 10.3, and 11.2 years, respectively; *p* = 0.03) and were less likely to be exposed to passive smoking (11%, 37%, and 29%, respectively; *p* = 0.001). Otherwise, characteristics did not differ significantly among the exposure groups.

**Table 2:** General characteristics of the study population in the Three Gorges Reservoir Region.^*a*^

Parameter	Unexposed children	Low-exposed children	High-exposed children	*p-*Value
No. (%)		145 (11)		183 (14)		994 (75)		–
Male:female (%)		52:48		54:46		51:49		0.75*b*
Age (years)		11.2 ± 1.8		10.3 ± 0.7		11.6 ± 2.5		0.03*c*
HBV infection (*n*)		3		4		37		0.39*b*
Hepatotoxic medicines used (*n*)		7		9		65		0.55*b*
Consumption of water per child per day (L)		1.1 ± 0.5		1.2 ± 0.6		1.1 ± 0.4		0.09*c*
Strenuous exercise [yes:no (*n*)]		18:127		28:155		162:832		0.48*b*
BMI (*n*)								0.23*b*
< 16		39		49		288		
16–18		51		62		391		
> 19		55		72		315		
Frequency of aquatic food consumption (*n*)								0.39*b*
< 1 time/week*d*		12		11		81		
1–2 times/week		73		89		487		
3–6 times/week		47		65		367		
> 6 times/week		13		18		59		
Smoking status (*n*)								0.001*b*
Nonpassive smoker		102		116		878		
Passive smoker		42		67		110		
Smoker		1		0		8		
**a**Values are mean ± SD except where noted. **b**From chi-square test. **c**From Kruskal–Wallis test. **d**Intake of child = 25 g aquatic food each time.								

*Assessment of total daily intake of microcystin in children.* According to our questionnaires, average water consumption was 1.2 L/child/day (Table 3). The mean MC-LR concentrations of untreated water samples collected from lake 1 and lake 2 during 5 years were 0.24 µg/L and 2.58 µg/L, respectively. Based on the reports of Nicholson et al. (1994), water treatment facilities remove approximately 40% of microcystin through chlorination. Therefore, we estimated that actual MC-LR intakes from drinking treated lake water per child per day were 0.14 μg and 1.55 μg for low- and high-exposed children, respectively. On average, each child consumed local fish or duck three times a week, for an average intake of 11 g wet weight or 2.2 g dry weight per day. The mean microcystin concentrations in lyophilized (freeze-dried) fish and duck muscle samples were 0.10 µg/g and 0.22 µg/g for lake 1 and lake 2, respectively. Thus, we estimated daily microcystin intakes from aquatic food of 0.22 μg or 0.48 μg and total daily intakes of 0.36 µg and 2.03 µg for low- and high-exposed children, respectively (Table 3). The latter value was much higher than the TDI of 0.4 µg for a 10-kg child per day as proposed by the WHO (1999).

**Table 3:** Estimated average total daily intakes of microcystin in exposed children in Three Gorges Reservoir Region.

Exposure	Low exposed	High exposed
Through drinking water				
Microcystin concentration in water (mean)*a*		0.24		2.58
Daily water intake/child (L)		1.2		1.2
Microcystin daily intake by drinking water/child (µg)		0.14		1.55
Through aquatic food				
Microcystin concentration in aquatic food (mean in lyophilized muscle)*b*		0.10		0.22
Daily aquatic food intake/child [dry (g)]		2.2		2.2
Microcystin daily intake by food/child (µg)		0.22		0.48
Total daily intake (drinking water + aquatic food)/child (µg)		0.36		2.03
WHO TDI of microcystin for 10-kg (BW) child (µg)		0.4		
BW, body weight. **a**Micrograms MC-LReq per liter. **b**Micrograms MC-LReq per gram.				

*Potential risk factors for liver damage.* We investigated six possible risk factors for liver damage by using multivariable logistic regression analysis (Table 4). HBV infection was the strongest risk factor [odds ratio (OR) 7.5; 95% confidence interval (CI), 5.4–10.8], followed by use of hepatotoxic medicines (OR 3.5; 95% CI, 2.2–5.6) and microcystin exposure (OR 1.7; 95% CI, 1.1–2.8). Other factors were not clearly associated with liver damage.

**Table 4:** Risk factors for liver damage*a* as measured by serum enzyme levels in Three Gorges Reservoir Region.

Variable	OR (95% CI)		*p*-Value
Microcystin exposed		1.72	(1.05– 2.76)		0.03
HBV-positive*b*		7.59	(5.36–10.79)		0.001
Hepatotoxic medication use*c*		3.49	(2.17–5.63)		0.001
BMI					
< 16		1.0	—		—
16–18		1.03	(0.24–2.88)		0.33
> 18		1.02	(0.22–2.65)		0.39
Strenuous exercise*d*		0.85	(0.19–2.36)		0.48
Passive smoking exposure*e*		1.48	(0.74–3.15)		0.13
**a**Liver damage (dichotomous based on two or more abnormally elevated liver enzyme assays). **b**HBV infection (yes or no). **c**Hepatotoxic medicine use (yes or no). **d**Strenuous exercise the previous day (yes or no). **e**Passive smoking (yes if exposed to environmental smoke > 15 min/day and > 1 day/week; no otherwise).					

*Serum liver damage enzymes levels.* Table 5 presents the results of liver damage enzyme levels in children. After adjustment for HBV infection and use of hepatotoxic medicines, AST (*p* = 0.01) and ALP (*p* = 0.001) were significantly increased in the high-exposed children (AST = 33.4 U/L, ALP = 288.7 U/L) compared with the low-exposed children (AST = 30.3 U/L, ALP = 285.6 U/L) and the unexposed children (AST = 27.6 U/L, ALP = 281.3 U/L). The proportion of children with at least one serum enzyme above the normal level was 10.8% in high-exposed children compared with 5.9% in low-exposed and 4.4% in the unexposed children. High-exposed children were more likely to be HBV infected (3.7%) than low-exposed children (2.2%) and unexposed children (2.1%).

**Table 5:** Serum liver damage enzyme levels in relationship with related risk factors in the three groups.

Risk factor	Unexposed (*n* = 145)	Low exposed (*n* = 183)	High exposed (*n* = 994)	*p*-Value
HBV infected [*n* (%)]		3 (2.1)		4 (2.2)		37 (3.7)		0.39*a*
Hepatotoxic medicines used [*n* (%)]		7 (4.8)		9 (4.9)		65 (6.5)		0.55*a*
HBV-negative, no hepatotoxic medicine use [*n* (%)]		135 (93.1)		170 (92.9)		892 (89.7)		0.22*a*
ALT (U/L)*b*		14.0 (12.6–15.4)		16.1 (14.5–17.7)		14.8 (14.2–15.4)		0.14*c*
AST (U/L)*b*		27.6 (26.2–29.0)		30.3 (29.3–31.3)		33.4 (32.8–34.0)		0.01*c*
GGT (U/L)*b,c*		14.8 (13.7–15.8)		17.0 (16.2–17.8)		16.2 (15.7–16.7)		0.18*c*
ALP (U/L)*b,c*		281.3 (269.5–293.1)		285.6 (272.9–298.3)		288.7 (281.4–296.0)		0.00*c*
Enzyme abnormalities [*n* (%)]		6 (4.4)		10 (5.9)		96 (10.8)		0.22*a*
**a**Chi-square test. **b**Values are 95% CI. **c**Kruskal–Wallis test.								

*MC-LR level in the serum.* To confirm MC-LR internal exposure, we randomly selected approximately 50 participants from each of the three groups to test serum MC-LR concentrations (Table 6). The detection rates were 1.9%, 84.2%, and 91.9% in unexposed, low-exposed, and high-exposed children, respectively; the mean concentration in the high-exposed children was 1.3 μg MC-LReq/L higher (*p* = 0.04) than in the low-exposed children (0.4 μg MC-LReq/L) and unexposed children (< LOD).

**Table 6:** Serum MC-LR levels according to exposure group.

MC-LR detection	Unexposed	Low exposed	High exposed	*p-*Value
Serum samples detected (*n*)		54		57		62		–
MC-LR–positive [*n* (%)]		1 (1.9)		48 (84.2)		57 (91.9)		0.001*a*
Mean (mean ± SD)*b*		< LOD*c*		0.4 ± 0.1		1.3 ± 0.2		0.04*d*
**a**Chi-square test. **b**Micrograms MC-LReq/L. **c**LOD = 0.1 μg/L. **d**Kruskal–Wallis test.								

## Discussion

The three sites we selected in this study showed dramatically different MC-LR concentrations both in water and aquatic food samples. The highest MC-LR concentration was observed in lake 2, a closed lake (not connected to a river) that has been used extensively for aquaculture. The highest annual mean concentration was up to 4.3 ± 1.0 μg MC-LReq/L, four times higher than the limit of 1.0 μg/L in water proposed by the WHO (1999). The owner of lake 2 stopped using the lake for aquaculture in 2009, resulting in a sharp reduction of MC-LR concentrations (Table 1), but consumers had been exposed to high MC-LR levels for many years before that. Lake 1 is fed by the Yangtze River, so microcystis blooms were uncommon, and we estimated that MC-LR concentrations would have been under the limit of 1.0 μg/L proposed by WHO after chlorination treatment. As expected, the lowest MC-LR concentrations were found in well water, with most samples below the LOD (0.1 μg/L). The detection of microcystin in well water in 2008 coincided with a heavy rain that lasted 4 days, which may have resulted in contamination of the wells by rainwater.

The analysis of fish and ducks from the lakes showed a 100% positive rate of microcystin, with the highest concentration in *A. nobilis* followed by *H. molitrix* and *C. carpio*. Because fish and duck are the most common aquatic food in Three Gorges Reservoir Region, the high microcystin detection frequencies in these food samples suggest that eating aquatic food from contaminated water might pose a health risk for frequent consumers. In our study, passive smoking appears to have no association with liver damage, which is consistent with some studies (Chavez et al. 2006) but not others (Pelucchi et al. 2008).

We investigated serum enzyme levels among children exposed to microcystin through drinking water and consuming aquatic food in the Chongqing area of the Three Gorges Reservoir Region. Mean AST and ALP values were higher in microcystin-exposed children than in unexposed children. Similar results have been reported in Australia, where levels of ALT and GGT in a presumed microcystin-exposed group were significantly higher than those in an unexposed group (Falconer et al. 1983). In another study, chronic exposure to microcystin in fishermen was followed for > 5 years, and microcystin concentrations showed closer positive relationships with ALT, AST, GGT, and ALP than with other serum enzymes (e.g., blood urea nitrogen, cholinesterase, albumin), suggesting that microcystin accumulation might have influences on these serum enzymes that reflect liver function (Chen et al. 2009a).

There are limited epidemiologic data in terms of impact on serum enzymes by microcystin exposure. In a study by Chen et al. (2009a), the proportion of fishermen with abnormal serum enzymes was 31% (11 of 35), approximately three times higher than the proportion of high-exposed children with abnormal liver enzymes in our study population (10.8%; 96 of 892) and six times greater than the proportion of low-exposed children (5.9%; 10 of 170). Chen et al. (2009a) excluded positive people from the fishermen, but did not control for other risk factors. In addition, the adult fisherman in the study by Chen et al. (2009a) probably would have been exposed for a much longer time than the children in our study.

Yu (1995) reported that populations who used pond-ditch water with microcystin pollution as their drinking water source experienced higher liver carcinoma mortality rates than populations that drank well water, and they pointed out that microcystin pollution in pond-ditch water was one of the risk factors for the high incidence of primary liver cancer in China. In our study, 0.9% of parents of high-exposed children self-reported a cancer diagnosis (9 of 994, including four with hepatic carcinoma) compared with 0.5% of parents of low-exposed children (1 of 183) and none of the parents of children in the unexposed group.

Our study has several limitations. First, some parents of participants reported that they were infected with hepatitis A virus, but we did not test for hepatitis A virus in any of the participants. Second, we tested for MC-LR in drinking water and aquatic food only, which may underestimate the overall exposure of microcystin. Finally, in multivariable logistic regression, the exercise measure used is a very crude indicator of exercise level, so this may affect the accuracy of the data.

## Conclusion

The detection of microcystin in drinking water, food, and serum of the study children, combined with the results of regression analysis, supports the hypothesis that microcystin exposure was associated with increased serum enzyme levels and liver damage among the study children. This conclusion may help governments enact relevant policy to prohibit aquaculture in lakes that supply drinking water. Once water or food sources are found to be contaminated by algae or microcystin, they should no longer be consumed. Furthermore, development of a more sensitive microcystin detection method and a search for more appropriate biomarkers in exposed populations should be pursued in future research studies.
